# Evaluation of HOTAIRM1, miR-196b, and HOXA9 as Oncogenic Markers in Patients with Acute Myeloblastic Leukemia 

**DOI:** 10.30699/ijp.2025.2030358.3309

**Published:** 2025-07-01

**Authors:** Fahime Norozi, Mehdi Allahbakhshian, Nader Vazifeshiran, Zahra Hasanpour, Mohsen Hamidpour

**Affiliations:** 1 Department of Hematology and Blood Banking, School of Allied Medical Sciences, Shahid Beheshti University of Medical Sciences, Tehran, Iran; 2 HSC Research Centre, Department of Hematology and Blood Banking, School of Allied Medical Sciences, Shahid Beheshti University of Medical Sciences, Tehran, Iran

**Keywords:** Lung cancer, Adenocarcinoma, Squamous cell carcinoma, TTF-1, Napsin A, p40, p63

## Abstract

**Background & Objective:**

miR-196b, HOXA9, GFI1, and PIM1 are key factors involved in cellular signaling pathways that contribute to the pathogenesis of malignancies, including acute myeloblastic leukemia (AML). Given their critical roles in AML progression, the present study aimed to investigate the gene expression levels of HOTAIRM1, miR-196b, HOXA9, GFI1, and PIM1 in AML patients compared to healthy controls.

**Methods:**

A total of 30 AML patients and 10 healthy volunteers were enrolled in this study. Peripheral blood and bone marrow mononuclear cells were isolated using Ficoll-Paque density gradient centrifugation. Gene expression levels of HOTAIRM1, miR-196b, HOXA9, GFI1, and PIM1 were assessed using real-time quantitative PCR (RQ-PCR). Statistical analyses were performed using Student’s t-test, one-way ANOVA, and Pearson correlation tests.

**Results:**

The expression levels of HOTAIRM1, miR-196b, HOXA9, and GFI1 were significantly elevated in AML patients compared to healthy controls. Furthermore, t-test analysis revealed that the expressions of HOTAIRM1, HOXA9, and GFI1 significantly differed between AML-M3 and non-M3 AML subtypes.

**Conclusion:**

These findings suggest that the investigated markers, particularly HOTAIRM1, HOXA9, and GFI1, may serve as potential clinical biomarkers for monitoring AML progression and could be valuable targets for early detection or therapeutic intervention.

## Introduction

Acute myeloid leukemia (AML) is the most common type of acute leukemia in adults and is a heterogeneous, invasive hematologic malignancy characterized by excessive proliferation of immature myeloid cells and impaired differentiation (1,2). Both genetic and epigenetic alterations contribute to AML pathogenesis. Among these, proteins and non-coding RNAs—notably microRNAs (miRNAs) and long non-coding RNAs (lncRNAs)—play crucial roles. MicroRNAs, typically 18–25 nucleotides in length, and lncRNAs, which are longer than 200 nucleotides, are involved in regulating gene expression at multiple levels and significantly influence the behavior of malignant cells, including their response to therapy in AML (3–5).

In this context, HOTAIRM1, miR-196b, HOXA9, GFI1, and PIM1 are five molecular markers implicated in AML progression.

HOTAIRM1, a myeloid-specific lncRNA, modulates HOXA cluster gene expression and plays a pivotal role in myelopoiesis (6). Its overexpression has been associated with increased diagnostic value, aggressiveness, drug resistance, and immune evasion in AML (7,8).

miR-196b functions as a negative regulator of granulocytic differentiation and enhances leukemic cell proliferation, survival, and resistance to apoptosis (9). High miR-196b expression is observed in AML cases with MLL rearrangements, FLT3-ITD, and NPM1 mutations, while low expression is reported in CEBPA-mutated AML (10–12).

HOXA9, a homeodomain-containing transcription factor, plays a key role in hematopoietic stem cell expansion (13). Overexpressed in more than 50% of AML cases, HOXA9 is considered a strong predictor of poor prognosis. It promotes proliferative gene expression and suppresses genes involved in myeloid differentiation and inflammatory responses (14,15).

GFI1 is essential for granulocytic differentiation (16) and regulates key cellular mechanisms, including lineage determination between granulocytes and monocytes, as well as lymphopoiesis of B cells, T cells, and dendritic cells (17,18).

PIM1 is a serine/threonine kinase regulated by the JAK/STAT pathway. It is involved in cell cycle progression—through inhibition of p21, p27, and CDC25A, and stimulation of NuMA—as well as apoptosis inhibition by upregulating Myc and Bcl-2 and suppressing BAD and ROS (19–21). Elevated expression of PIM1 has been reported in AML subtypes, particularly those with FLT3-ITD mutations (22).

Given the prominent roles of HOTAIRM1, miR-196b, HOXA9, GFI1, and PIM1 in AML pathogenesis, this study aimed to evaluate the expression profiles of these genes and examine their correlation with clinical characteristics in AML patients.

## Materials and Methods

### Patients

This case-control study was conducted on 30 newly diagnosed AML patients and 10 healthy volunteers as the normal control group at Taleghani Hospital (Tehran) during the years 2021–2022. The study protocol was approved by the Ethics Committee of Shahid Beheshti University of Medical Sciences (IR.SBMU.RETECH.REC.1400.691), and informed consent was obtained from all participants in accordance with the Declaration of Helsinki.

### Sample Preparation

Samples were collected from the bone marrow (BM) and peripheral blood (PB) of both patients and controls. White blood cells (WBCs) from bone marrow and peripheral blood were isolated separately. The phenotype of blast cells was determined using flow cytometry (FACS, ABI, USA). Demographic and clinical characteristics of patients, including age, gender, sample type, AML subtype, and blast percentage, are presented in Table 1. Mononuclear cells were separated using Ficoll-Paque density gradient centrifugation.

### RNA Extraction and cDNA Synthesis

Total RNA was extracted from mononuclear cells using an RNA extraction kit (Yekta Tajhiz Azma, Iran) according to the manufacturer’s protocol. To maximize RNA yield, especially microRNAs, the aqueous phase containing RNA was incubated overnight in isopropanol, followed by a final incubation in 100% ethanol. The integrity of the isolated RNA was verified by the presence of 28S and 18S ribosomal RNA bands on agarose gel electrophoresis. RNA quantity was assessed using a NanoDrop spectrophotometer (Thermo Scientific, USA). Complementary DNA (cDNA) was synthesized using the Easy cDNA Synthesis Kit (Iran) for lncRNA, mRNA, and microRNA targets. For microRNA reverse transcription, a stem-loop primer was employed due to the small size of microRNAs and their lack of poly-A tails.

### Quantitative Real-Time PCR

Gene expression levels of HOTAIRM1, miR-196b, HOXA9, GFI1, and PIM1 were measured using SYBR Green-based real-time PCR performed on a Rotor-Gene 6000 thermocycler (Qiagen, Germany). The total volume of each reaction mixture was 15 µL, consisting of 7.5 µL SYBR Green master mix (Amplicon, Denmark), 4.5 µL nuclease-free water, 1 µL of forward and reverse primers (Pishgam, Iran), and 2 µL of template cDNA. PCR thermal cycling included an initial denaturation step at 95°C for 10 minutes, followed by 40 cycles of denaturation at 95°C for 10 seconds, annealing at gene-specific temperatures for 15 seconds (63°C for HOTAIRM1, 59°C for miR-196b, 58°C for HOXA9, 64°C for GFI1, 63°C for PIM1, 64°C for ABL, and 62°C for U6), and a final extension at 72°C for 20 seconds. Melting curve analysis was performed to verify the specificity of each amplified product. ABL and U6 served as internal controls for normalization of mRNA/lncRNA and miRNA targets, respectively. The relative expression of each gene was calculated using the 2^–ΔΔCt (Livak) method. The primer sequences used for amplification are listed in Table 2.

### Statistical Analysis

All experiments were performed in duplicate. Statistical analysis was carried out using SPSS software (version 26). As the data followed a normal distribution, parametric tests including Student’s t-test and one-way ANOVA were used to evaluate differences in the expression levels of HOTAIRM1, miR-196b, HOXA9, GFI1, and PIM1 between AML patients and healthy controls. Pearson correlation analysis was applied to examine the relationships between gene expression levels.

## Results

**Fig. 1:**
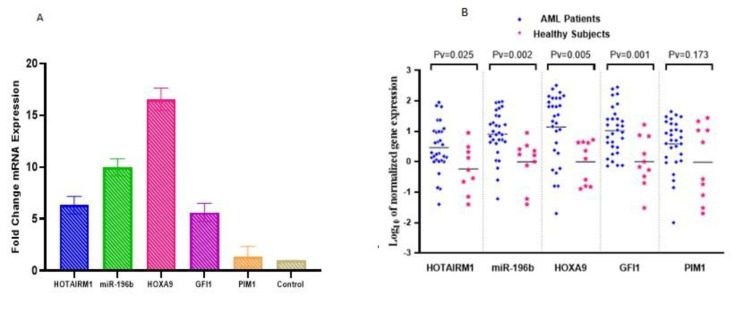
The HOTAIRM1, miR-196b, HOXA9, GFI1, PIM1 markers expression in AML.

The expression levels of HOTAIRM1, miR-196b, HOXA9, GFI1, and PIM1 were analyzed using real-time PCR in 30 AML patients and 10 healthy controls. The results showed that the expression levels of HOTAIRM1, miR-196b, HOXA9, and GFI1 were significantly upregulated in AML patients compared to the control group. Specifically, HOTAIRM1 showed a 6.35-fold increase (*P* < 0.05), miR-196b a 9.98-fold increase (*P* < 0.01), HOXA9 a 16.54-fold increase (*P* < 0.01), and GFI1 a 5.63-fold increase (*P* < 0.001). However, there was no significant difference in PIM1 expression between AML patients and healthy individuals (Fig. 1a and 1b).

**Table 1:** Demographic characteristics of patients

Blast (%)	Type Of Samples	Gender	Age	FAB	Patients
93	PB	M	48	AML-nonM3	1
97	PB	F	43	AML-nonM3	2
72	BM	M	39	AML-nonM3	3
80	BM	F	39	AML-nonM3	4
40	BM	M	48	AML-nonM3	5
70	BM	F	30	AML-nonM3	6
60	PB	F	92	AML-nonM3	7
55	BM	M	54	AML-nonM3	8
40	BM	F	60	AML-nonM3	9
35	BM	F	29	AML-nonM3	10
35	PB	M	74	AML-nonM3	11
65	BM	F	61	AML-nonM3	12
90	BM	M	46	AML-nonM3	13
50	BM	M	38	AML-nonM3	14
80	BM	F	34	AML-nonM3	15
95	PB	F	26	AML-nonM3	16
85	PB	M	78	AML-nonM3	17
80	PB	M	25	AML-nonM3	18
50	BM	M	25	AML-nonM3	19
45	BM	M	64	AML-nonM3	20
70	BM	F	58	AML-M3	21
35	BM	F	84	AML-nonM3	22
35	BM	F	26	AML-M3	23
90	PB	M	61	AML-nonM3	24
80	BM	F	34	AML-nonM3	25
85	PB	M	78	AML-nonM3	26
50	BM	M	25	AML-nonM3	27
45	BM	M	64	AML-nonM3	28
75	PB	F	57	AML-M3	29
90	PB	M	61	AML-nonM3	30

**Table 2:** Marker name, primer sequence, and amplified fragment length for all five markers

markers	Forward primer (5′-3′)	Reverse primer (5′ − 3)	Amplicon Size (bp)
HOXA9	CTG ACT ATG CTT GTG GTT CTC	TGG CTG CTG GGT TAT TGG	132
GFI1	AGA CCC TTT GCC TGC GAG ATG	TAC AGT CAA AGC TCC GTT CCT G	100
PIM1	GCT CAA GGA CAC CGT CTA CAC	GAG ATG CTG ACA TTC TGA AGA GAC	223
HOTAIRM1	AGG GGG TTG AAA TGT GGG TG	CTT GAA AGT GGA GAA ATA AAG TGC C	162
ABL	CTT CTT GGT GCG TGA GAG TGA G	GAC GTA GAG CTT GCC ATC AGA AG	115
miR-196b Forward	GTG CTA GGT AGT TTC CTG TTG	-	-
U6 Forward	GGG CAG GAA GAG GGC CTA T	-	-
Universal Reverse	GAG CAG GGT CCG AGG T	-	-
Stem Loop	GGTCGTATGCAGAGCAGGGTCCGAGGTATCCATCGCACGCATCGCTCTGCATACGACCCCCAA	-	-

**Table 3:** The correlation among expressions of HOTAIRM1, miR-196b, HOXA9, GFI1, and PIM1 in AML patients

Markers	HOTAIRM1		miR-196b		HOXA9		GFI-1		PIM-1	
	P-value	r	P-value	r	P-value	r	P-value	r	P-value	r
HOTAIRM1	-	1	0.003	0.529^**^	0.002	0.554^**^	0.071	0.335	0.247	0.222
miR-196b	0.003	0.529^**^	-	1	0.000	0.819^***^	0.731	0.065	0.414	0.158
HOXA9	0.002	0.554^**^	0.000	0.819^***^	-	1	0.782	0.054	0.797	0.051
GFI1	0.071	0.335	0.731	.065	0.782	0.054	-	1	0.069	0.342
PIM1	0.247	0.222	0.414	0.158	0.797	0.051	0.069	0.342	-	1

**A**, The expression level of HOTAIRM1, miR-196b, HOXA9, GFI1 increased in AML patients compared with controls (HOTAIRM1: 6.35-fold (P < 0.05), miR-196b: 9.98-fold (P < 0.01), HOXA9: 16.54-fold (P < 0.01), GFI1: 5.63-fold (P < 0.001)). There was no significant difference in the expression of PIM1 between AML patients and controls.

**B,** The comparison of HOTAIRM1, miR-196b, HOXA9, GFI1, and PIM1 expression between AML patients and controls

**Fig. 2:**
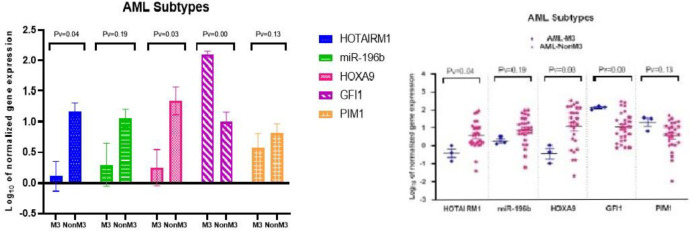
The differences among the gene expressions of HOTAIRM1, miR-196b, HOXA9, GFI1, and PIM1 in AML subtypes (AML-M3, AML-nonM3).

The results show that the expression level of HOTAIRM1 and HOXA9 increased in AML-nonM3 in comparison with AML-M3 (HOTAIRM1: P < 0.05, HOXA9: P < 0.05), and the expression level of GFI1 is significantly increased in AML-M3 in comparison with AML-nonM3 (P < 0.001), There was no significant difference in the expression of miR-196b and PIM1 between AML-M3 and AML-nonM3.

To examine whether expression levels of these markers varied by AML subtype, patients were classified into two groups: AML-nonM3 and AML-M3, based on molecular and cytogenetic analysis. T-test analysis revealed that HOTAIRM1 (*P* < 0.04) and HOXA9 (*P* < 0.03) expression levels were significantly higher in the AML-nonM3 group compared to the AML-M3 group. Conversely, GFI1 expression was significantly elevated in the AML-M3 group compared to AML-nonM3 (*P* < 0.01). No significant differences were observed in the expression of miR-196b (*P* < 0.19) or PIM1 (*P* < 0.13) between these two AML subtypes (Fig. 2).

**Fig. 3:**
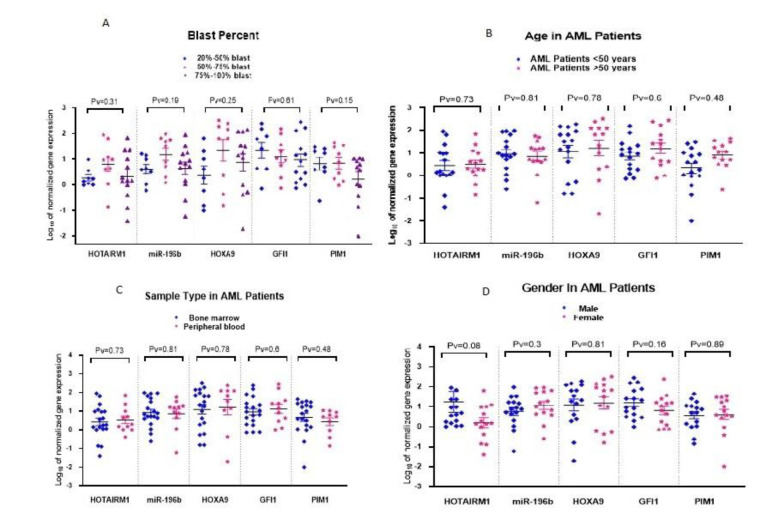
**a.** The comparison of HOTAIRM1, miR-196b, HOXA9, GFI1, and PIM1 expression according to the three blast percentage groups (20-50, 50-75, and 75-100). There was no significant difference in the expression of HOTAIRM1, miR-196b, HOXA9, GFI1, and PIM1 among these three blast percentage groups.

To explore potential correlations between marker expression and demographic or clinical parameters, expression levels were compared across gender, age groups (<50 vs. ≥50 years), sample type (bone marrow vs. peripheral blood), AML subtypes, and blast percentages (20–50%, 50–75%, 75–100%). Expression levels were analyzed using the t-test for binary comparisons and ANOVA for multi-group comparisons. No statistically significant associations were found between any of the gene expression levels and these demographic or clinical characteristics (Fig. 3a–d).

Furthermore, Pearson correlation analysis was performed to assess potential relationships among the expression levels of HOTAIRM1, miR-196b, HOXA9, GFI1, and PIM1. A strong and statistically significant positive correlation was observed among HOTAIRM1, miR-196b, and HOXA9 expression in AML patients. The strongest correlation was identified between HOXA9 and miR-196b (*P* = 0.00, *r* = 0.819) (Table 3).

Additionally, analysis of blast percentage subgroups revealed that as the blast percentage increased (from 50% to 75%), the correlation among HOTAIRM1, miR-196b, and HOXA9 also strengthened. The strongest correlation was observed in the 75–100% blast group, where the association between HOXA9 and miR-196b reached *P* = 0.00 and *r* = 0.945.

**b.** The comparison of HOTAIRM1, miR-196b, HOXA9, GFI1, and PIM1 according to the two aging groups (less and more than 16 years old). There was no significant difference in the expression of HOTAIRM1, miR-196b, HOXA9, GFI1, and PIM1 between the two aging groups.

**c.** The comparison of HOTAIRM1, miR-196b, HOXA9, GFI1, and PIM1 according to the two sample groups (Bone marrow and peripheral blood). There was no significant difference in the expression of HOTAIRM1, miR-196b, HOXA9, GFI1, and PIM1 between the two sample groups.

**d.** The comparison of HOTAIRM1, miR-196b, HOXA9, GFI1, and PIM1 according to the two gender groups (male and female). There was no significant difference in the expression of HOTAIRM1, miR-196b, HOXA9, GFI1, and PIM1 between the two gender groups.

## Discussion

Acute myeloid leukemia (AML) is the most common type of acute leukemia in adults, accounting for approximately 80% of cases (2). Hematopoietic stem cell differentiation into mature blood cells is a multi-step process tightly regulated by a set of myeloid-lineage transcription factors. In various malignancies, alterations in gene expression profiles can lead to uncontrolled cell proliferation. In addition to transcription factors, non-coding RNAs—especially long non-coding RNAs (lncRNAs) and microRNAs (miRNAs)—play critical roles in regulating gene expression and have been increasingly implicated in hematologic cancers, including AML (4, 23).

In this study, we investigated the expression levels of five oncogenic markers—HOTAIRM1, miR-196b, HOXA9, GFI1, and PIM1—in AML patients using real-time PCR. Our results showed a significant upregulation of HOTAIRM1 (*P* < 0.05), miR-196b (*P* < 0.01), HOXA9 (*P* < 0.01), and GFI1 (*P* < 0.001) in AML patients compared to healthy controls. In contrast, PIM1 expression was not significantly different between the two groups.

Previous studies have shown that HOTAIR, a related lncRNA, is associated with multiple cancers, including head and neck, ovarian, gastric, hepatic, colorectal cancers, and glioblastoma (24–26). Similarly, high HOTAIRM1 expression has been linked to intermediate-risk AML and correlated with reduced overall and leukemia-free survival, as well as increased relapse risk (27). Esmaili et al. reported HOTAIR overexpression in AML patients and demonstrated a positive correlation with STAT3, suggesting that HOTAIRM1 may promote leukemic proliferation via STAT3 signaling (28).

HOTAIRM1 has also been implicated in drug resistance in AML, potentially through the activation of glycolytic and Wnt/β-catenin signaling pathways, which influence tumor metabolism and progression (29). Additional evidence suggests that HOTAIR can induce drug resistance through miR-203a-3p–mediated regulation of β-catenin and GRG5 (30). Because dendritic cells play a critical role in antitumor immunity, upregulation of HOTAIRM1 may impair immune responses and reduce the effectiveness of immunotherapies (8). In our study, higher expression of HOTAIRM1 was observed predominantly in the AML-nonM3 subgroup.

The role of miR-196b has been demonstrated in multiple malignancies, including lung, oral, gastric cancers, as well as acute lymphoblastic leukemia (ALL) and chronic myeloid leukemia (CML) (31–34). Overexpression of miR-196b has been associated with intermediate-risk AML (27), and is frequently observed in AML with MLL rearrangements and FLT3-ITD mutations, which are indicators of poor prognosis. Conversely, lower expression of miR-196b has been reported in AML with CEBPA mutations, which are typically associated with better outcomes (10, 11). Based on these findings, miR-196b appears to play a role in promoting proliferation and survival in AML, including the M3 subtype.

Our results also revealed significant overexpression of HOXA9 in AML patients, particularly in the AML-nonM3 subgroup, supporting its role as a potent oncogene. HOXA9 overexpression has been shown to induce AML in murine models within 3 to 10 months and suppress differentiation markers such as CD11b (35, 36). Dysregulated HOXA9 expression is associated with genetic abnormalities including NPM1c mutations, MLL rearrangements, CDX deregulation, NUP98 fusions, and monocytic leukemia zinc finger (MOZ) fusions (37, 38).

GFI1 expression was also significantly elevated in AML patients, with higher levels observed in AML-M3 compared to AML-nonM3. This finding aligns with previous studies indicating that GFI1 acts as a tumor suppressor. Reduced expression of GFI1 has been shown to contribute to AML progression in animal models (39), while its overexpression has been associated with better prognosis and improved overall survival in AML patients (40). Thus, GFI1 may play a dual role, depending on the cellular context and AML subtype.

PIM1, a serine/threonine kinase, is known to be overexpressed predominantly in hematologic malignancies and is a downstream target of HOXA9, especially in AML with FLT3-ITD mutations (19, 42). However, in our study, PIM1 expression did not significantly differ between AML patients and controls. This may be due to a low prevalence of FLT3-ITD mutations in our sample population. Moreover, PIM1 has been reported to be upregulated in advanced AML stages but not in early or untreated blasts (44). This discrepancy might explain the variable findings across different studies and highlights a potential limitation of our study due to sample heterogeneity and mutation frequency.

Although mutations in PIM1 are believed to be oncogenic, definitive conclusions about their role in patient prognosis remain elusive. Bellon et al. suggested a potential but unconfirmed association between PIM gene alterations and decreased survival in AML (41). PIM1 is also involved in cellular homing and migration, processes that contribute to FLT3-ITD–mediated leukemogenesis (43). Further investigation into the role of PIM kinases in distinct AML subtypes is warranted (44, 45).

In summary, our findings show significant upregulation of HOTAIRM1, miR-196b, HOXA9, and GFI1 in AML patients compared to healthy controls. Differential expression patterns were observed between AML-M3 and AML-nonM3 subtypes: HOTAIRM1, miR-196b, and HOXA9 were predominantly overexpressed in AML-nonM3, whereas GFI1 was more elevated in AML-M3. Furthermore, a strong positive correlation was found among the expression levels of HOTAIRM1, miR-196b, and HOXA9, with the most robust association between HOXA9 and miR-196b.

These results support the potential of these biomarkers for subtype differentiation, prognosis, and therapeutic targeting in AML. However, additional studies with larger, genetically characterized cohorts are needed to further elucidate their roles in leukemogenesis and to validate their clinical utility.

## Conclusion

Based on the findings of this study, HOTAIRM1, miR-196b, and HOXA9 appear to play oncogenic roles in AML. Their involvement in key cellular processes such as differentiation, proliferation, and apoptosis suggests that their overexpression contributes to leukemogenesis and disease progression. Targeted repression of these markers may represent a promising future therapeutic strategy for preventing or controlling AML progression.
